# Leaf beetle decline in Central Europe (Coleoptera: Chrysomelidae s.l.)?^*^

**DOI:** 10.3897/zookeys.856.32564

**Published:** 2019-06-17

**Authors:** Angelique Wendorff, Michael Schmitt

**Affiliations:** 1 Ernst-Moritz-Arndt-Universität Greifswald, Allgemeine & Systematische Zoologie, Loitzer Str. 26, D-17489 Greifswald, Germany Ernst-Moritz-Arndt-Universität Greifswald Germany

**Keywords:** abundance, collection data, geographical distribution, insect decline

## Abstract

Based on 168,674 records in the database ChryFaun changes in distribution and abundance of leaf beetles (Chrysomelidae s.l.) in Central Europe were analysed from 1900 through 2009. From the first decade (1900–1909) to the last (2000–2009) the number of records per decade increased by factor 26, from 1513 to 41,269. The number of species increased from 395 in decade 1 to 606 in decade 10, but only 532 were reported in decade 11. The number of species with fewer records increased from 1990 although the total number of records increased continuously. Decrease and increase is found likewise in mono-, oligo-, and polyphagous species. Twenty-two species (3.0%) have not been reported since 1990, and 42 (5.8%) since 2000. 71% of all taxa reported between 2000 and 2009 had fewer records than in the immediately previous decade. These indications of decline correspond with numerous published studies on decline in other groups of arthropods. Analysis shows that data from private and public collections are useful for the retrospective analysis of numbers and distributions of leaf beetles (and other organisms).

## Introduction

The alarming news that the biomass of flying insects decreased by 75% in the course of the past 30 years (Hallmann et al. 2017) raised a remarkable public awareness of the general decline of biodiversity in Europe and elsewhere. Earlier studies (e.g., Thomas et al. 2004; Conrad et al. 2006; Kosior et al. 2007) had pointed in the same direction but were hardly noticed by the media and decision makers. Biesmeijer et al. (2006) had even demonstrated a parallel decline of pollinators and insect-pollinated plants in The Netherlands.

There is an ongoing controversy as to the causation of this process. Change in land use and intensified agriculture, loss or fragmentation of habitats, and the global climate change are considered as possible causes (see Conrad et al. 2006; Potts et al. 2010; Hallmann et al. 2017). The average temperature in Europe increased between 2006 and 2015 by 1.45–1.59 °C as compared to pre-industrial times (Kurnik 2017). Habitat fragmentation prevents individuals from natural dispersal so that local extinction events occur. As a consequence, smaller population sizes and a reduced ability to disperse of, e.g., *Cryptocephalusnitidulus* Fabricius, 1787 (Chrysomelidae: Cryptocephalinae) were observed in Britain (Piper and Compton 2010).

Changes in land use, habitats and climate certainly not only cause a decline of insects (and other organisms) but will also further range shifts and colonisation of new habitats as animals will track their preferred conditions if ever possible. In literature, we find numerous reports of an expansion or shift of ranges in beetles, butterflies, dragonflies, and grasshoppers to the north or to higher elevations (Parmesan 1996; Parmesan et al. 1999; Konvicka et al. 2003; Hickling et al. 2006), as well as spiders (Krehenwinkel and Tautz 2013) and birds (Thomas and Lennon 1999). Also leaf beetles seem to respond to increasing temperature by changing their distributional area, as shown for *Oulemamelanopus* (Linnaeus, 1758) in Canada (Olfert and Weiss 2006) and for *Leptinotarsadecemlineata* (Say, 1824) and *O.melanopus* in Europe (Svobodova et al. 2014).

We checked if decline and distributional change also occur in leaf beetles (Chrysomelidae s.l.) in Central Europe. To accomplish this we analysed the records in the database ChryFaun for the period from 1900 to 2009 or 2017. This database was compiled by the members of the working group “Faunistics of Central European leaf and seed beetles – ChryFaun”, founded in 1987 (Schmitt et al. 2014). We expected to find a number of species that extended or shifted their range northwards, and that the number of records for some species had decreased towards the end of our study period.

## Materials and methods

### The database

The database ChryFaun contains records from the end of the 19^th^ century to present, taken from museum and private collections, provided by institutions, individual amateur collectors, and regional entomological clubs (for details see Schmitt et al. 2014). Up to now (06.12.2018), 175,632 records for 726 species and 50 subspecies of Chrysomelidae sensu lato (i.e., including Megalopodidae, Orsodacnidae, and Bruchinae/Bruchidae) have been entered. We follow the nomenclature in Löbl and Smetana (2010).

**Figure 1.**
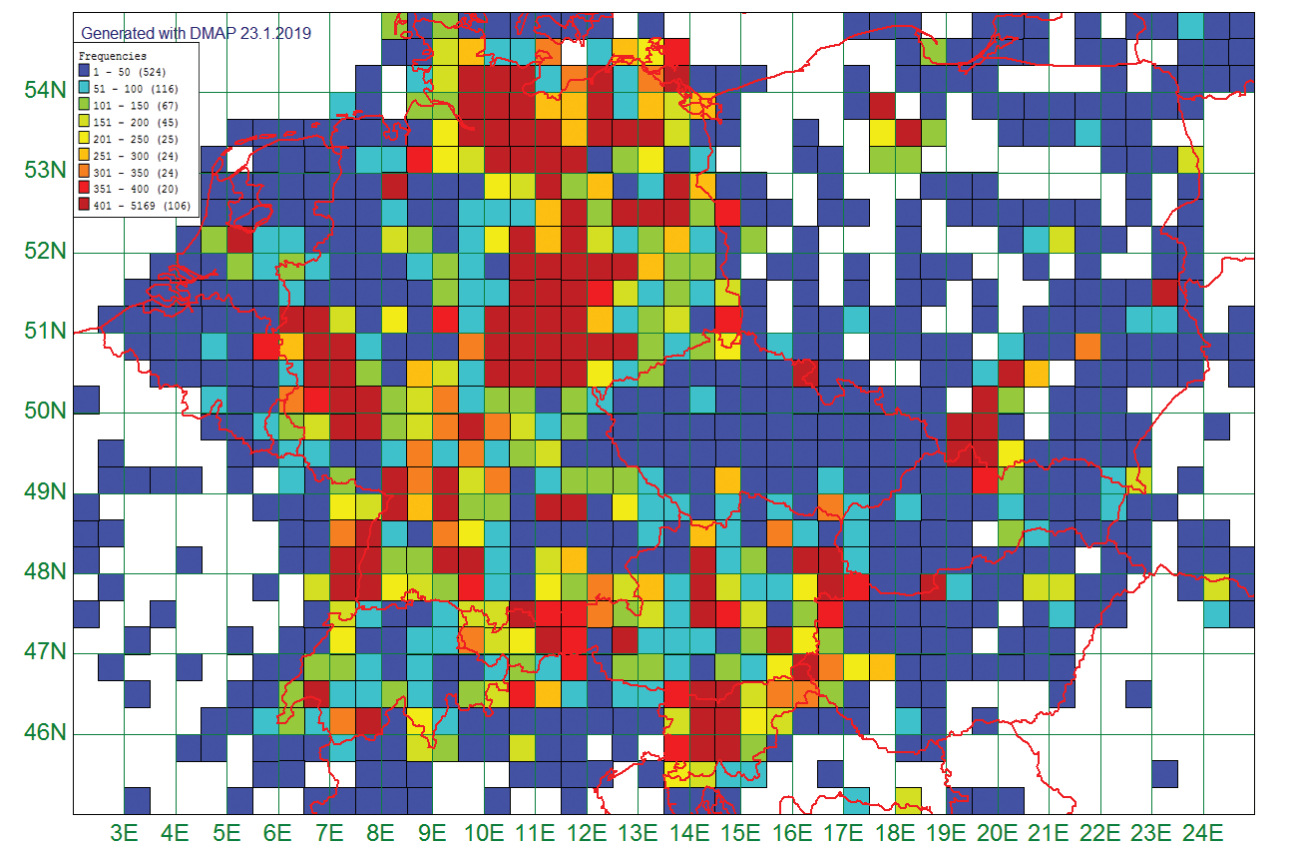
Distribution of the 175,632 records from 951 grid fields of 20 × 30 geographical minutes in Central Europe.

Operationally, “Central Europe” is defined as the rectangle between 2° and 25° eastern longitude and between 45° and 55° northern latitude. This area comprises The Netherlands, Belgium, Luxembourg, Germany, Switzerland, Liechtenstein, Austria, The Czech Republic, Slovakia, Poland, Hungary, Slovenia, and parts of France, Italy, Croatia, Serbia, Romania, Ukraine, Belarus, Russia, and Lithuania (see Fig. 1).

### Data analysis

Changes in distribution

We selected 246 species or subspecies out of the 776 taxa in ChryFaun. These are (1) species for which Schmitt and Rönn (2011) gave a northern, montane, southern, southeastern or southwestern distribution; (2) species for which we found an indication of distributional change in the literature; and (3) all additional species of the genera *Gonioctena*, *Orsodacne*, *Phyllotreta*, *Timarcha* and *Zeugophora*, as we suspected that they may be prone to behave ecologically similar to their congeneric species with ranges of the types listed under (1).

We divided the study period (1900 through 2017) into four quartiles, quartile 1: 1900–1929, quartile 2: 1930–1959, quartile 3: 1960–1989, and quartile 4: 1990–2017. We generated frequency maps of the distribution of all species studied for each quartile using the distribution mapping software DMAP (Alan Morton, Penrhyncoch, Aberystwyth, Ceredigion, UK – http://www.dmap.co.uk/, Version 7.4, 32-bit). Species with fewer than 24 records for the period from 1900 through 2017 were omitted.

We compared the four maps and recorded a change in distribution if the species extended or shifted its range from at least one quartile to the next for more than one degree latitude or/and longitude. We defined nine categories of change according to the direction of extension or shift: to the north, east, south, west, northeast, northwest, southeast, southwest, and “shrinking”. Since a species could extend or shift its range in more than one direction, we sorted some species to more than one category. We categorized a species distribution as “shrinking” when its range diminished, or when the species disappeared.

Increase or decrease of the number of records

Here, we considered the time period from 1900 through 2009 because we have too few entries for the last eight years and for the period prior to 1900. In the ChryFaun database are 165,506 records for the time period under study (as of January, 2019). The figures for each of the 11 decades were ascertained, and increase or decrease from each decade to the following was coded qualitatively and quantitatively. The proportion of species with de- and increased records per decade were calculated, their deviation from the mean was tested with Pearson’s Chi-squared test. We also tested the figures for mono-, oligo-, and polyphagous species separately. We performed χ²- and Fisher‘s exact tests using ‘R’ v. 3.4.3 (R Core Team 2017).

## Results

### Changes of distribution

We could not detect a change in 84 of the selected 246 taxa. The remaining 162 taxa fall in one or more than one of the described categories (Tab. 1).

**Table 1.** Change of distribution of 162 out of the 246 selected species of Chrysomelidae s.l. in central Europe (58 species are sorted into more than one category).

Change of distribution towards	Number of species
North	25
East	107
South	12
West	17
North-East	18
North-West	10
South-East	19
South-West	5
Shrinking	25

The complete list of species and their assignments are given in Appendix 1.

### Increase and decrease of reported records

The 175,632 records in ChryFaun from the time period end of 19^th^ century through 2017 are distributed unevenly over the area of Central Europe (Fig. 1). Approximately 114,500 records lie within Germany, with highest densities around Hamburg, in Thuringia, Saxony-Anhalt, in the Rhineland, and in the Alsace. Similarly high densities of records can be seen in eastern Austria around Lake Neusiedl, and also in the north and in the south of Poland. From some regions (white areas) we do not have records. Austria, Switzerland, Slovenia, and the major part of Germany are well covered.

**Figure 2.**
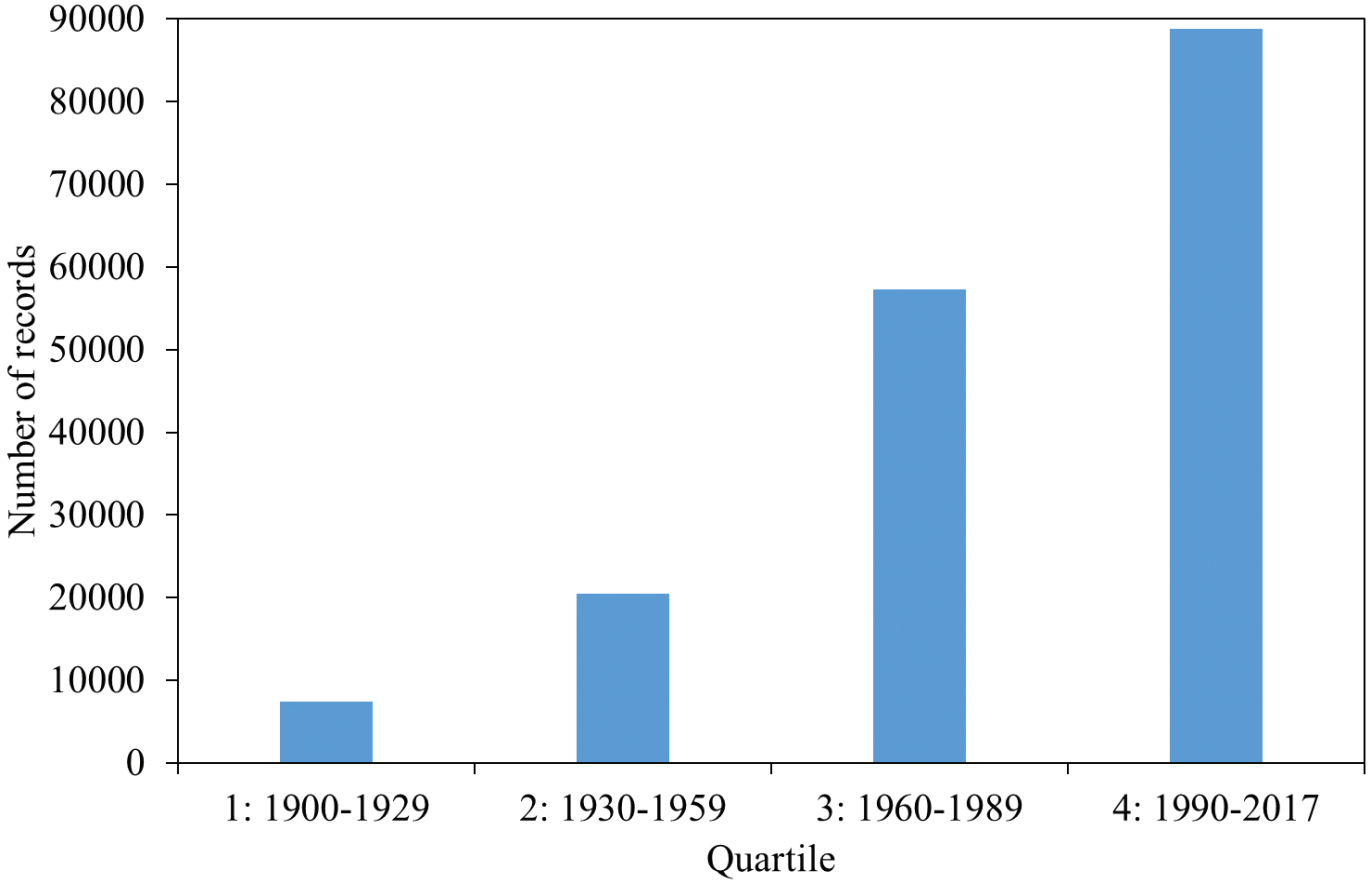
Numbers of records of Chrysomelidae s.l. in ChryFaun per quartile.

**Figure 3.**
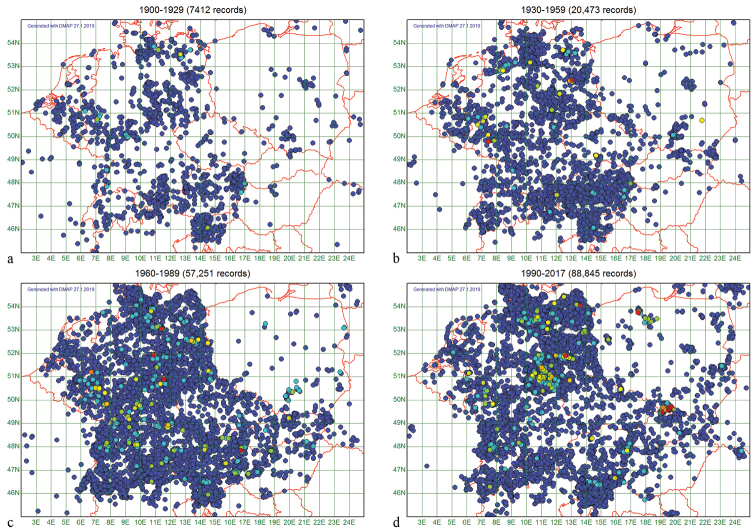
Geographical distribution of the records in ChryFaun for the four temporal quartiles shown as circles of 12.5 × 20 geographical minutes diameter. **a** 1900–1929 **b** 1930–1959 **c** 1960–1989 **d** 1990–2017.

We divided the study period into four quartiles, 1900–1929, 1930–1959, 1960–1989, 1990–2017, and identified the number of records for each quartile. The 173,981 records are distributed in a highly uneven manner, over time (Fig. 2) and in space (Fig. 3).

We have 7,412, 20,473, 57,251, and 88,845 records from quartile 1, 2, 3, and 4, respectively. The geographical distribution of the records (Fig. 3a–d) shows a similar pattern for each quartile in their overall distribution (Fig. 1).

We tested the figures of the four quartiles separately for species reported as monophagous, oligophagous, or polyphagous, respectively by Koch (1992). There were no significant differences in the proportions of species with de- or increased numbers of records from one quartile to the following.

**Figure 4.**
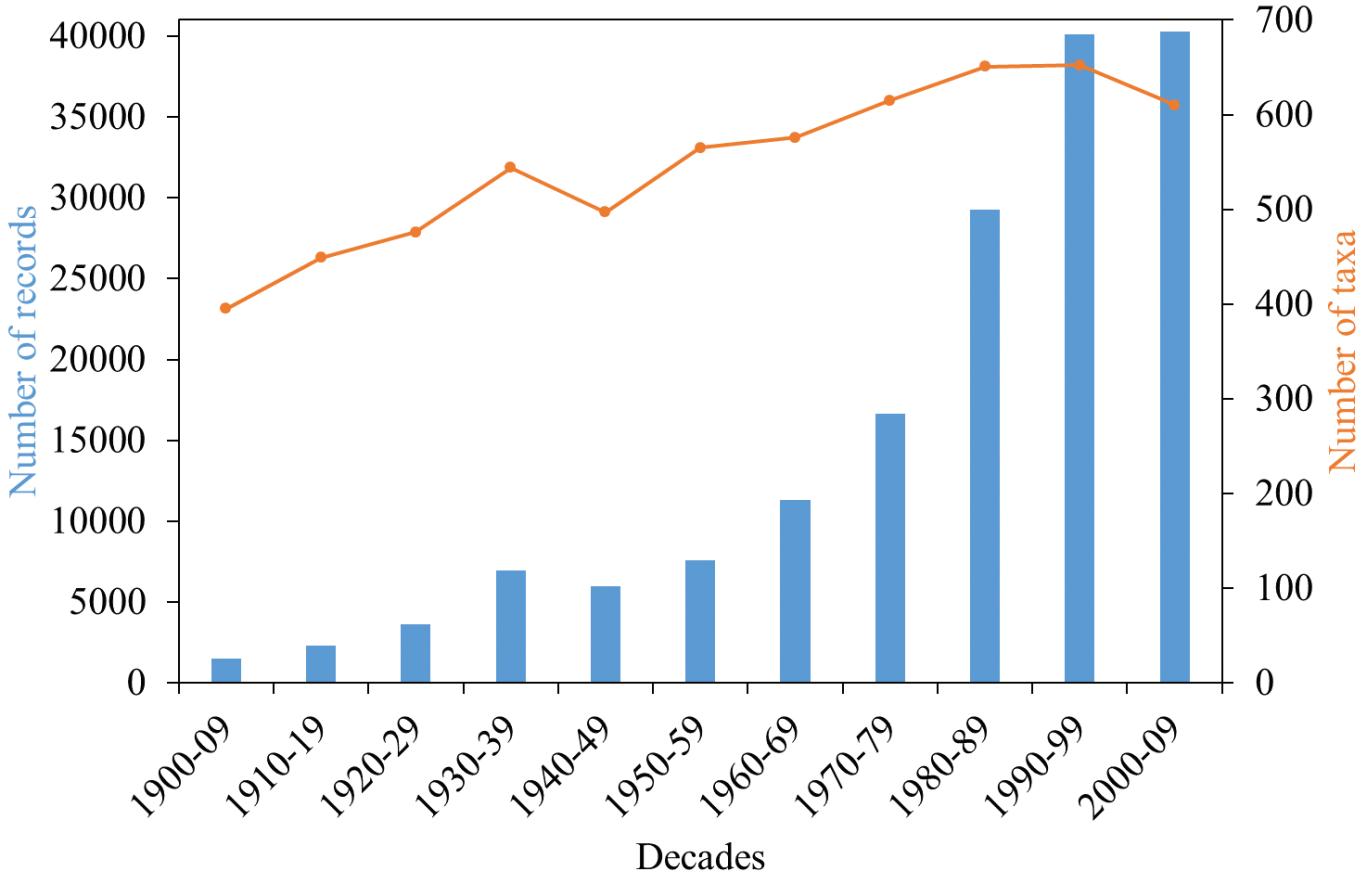
Number of records (blue columns) and number of reported taxa (species and subspecies -orange line) per decade between 1900 and 2009.

For more detailed analysis we listed the records per decade from 1900 through 2000. The time period was truncated at 2009 in order to compare full decades and because collectors often hand in their contributions with a delay. From decade 1(1900–1909) to decade 11 (2000–2009) the number of records in ChryFaun increased from 1513 to 40,269, i.e., by factor 26.6. This increase (± 0.5) is, however, caused by records that pertain to only three species: *Lochmaeacrataegi* (Forster, 1771), *Sclerophaedonorbicularis* (Suffrian, 1851), and *Chrysolinastaphylaea* (Linnaeus, 1758). In 229 taxa (species and subspecies) the increase is lower than by factor 26, and 19 taxa show an absolute decrease in records. The factor of increase is higher than 26 in only 123 taxa. For 402 of the 776 taxa we did not calculate such factors as either the numbers of their records were constant over the eleven decades or records were missing for decade 1 or decade 11. The number of reported taxa increased from 399 in decade 1 to 657 in decade 10, but only 616 were reported in decade 11 (Fig. 4, orange line).

**Figure 5.**
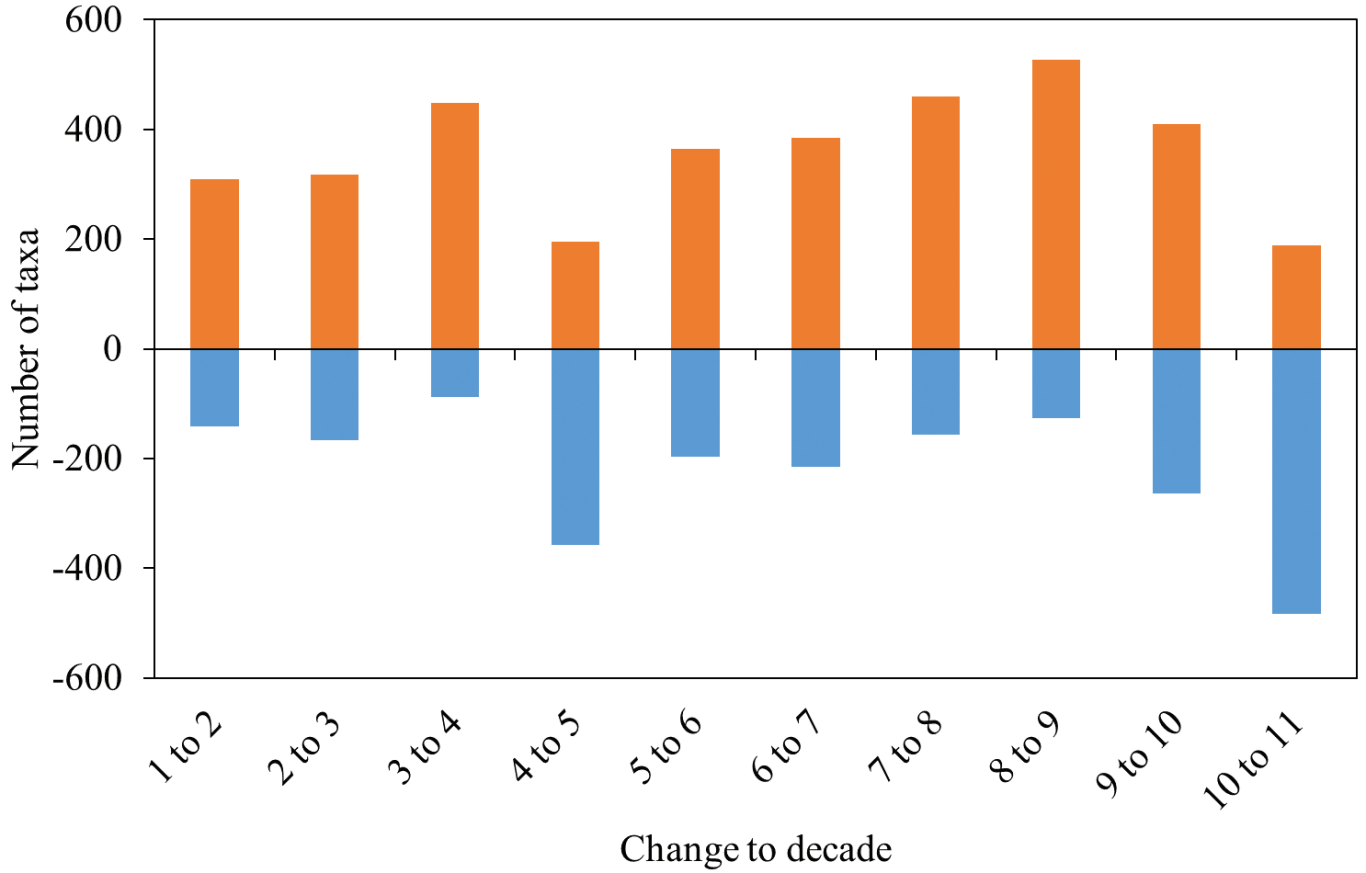
Number of taxa with an increase (orange) or a decrease (blue) of records from one decade to the following. The figures for increase and for decrease differ significantly from equal distribution (Pearson’s Chi-squared test: χ² = 195.18, df = 9, p-value < 2.2e-16 for the increase values, χ² = 323.05, df = 9, p-value < 2.2e-16 for the decrease values).

The number of species with increase or decrease from one decade to the following is not constant over time. There are significant deviations from equal distribution (increase: χ² = 195.18, df = 9, p-value < 2.2e-16, decrease: χ² = 323.05, df = 9, p-value < 2.2e-16, Pearson’s Chi-squared test, Fig. 5).

From decade 1 through decade 9 the number of those taxa with an increase in records (orange columns in Fig. 5) increases. At the same time, the number of taxa with a decrease of records (blue columns in Fig. 5) remains relatively constant, with the exception of the changes from decade 3 to 4 and from 4 to 5. Beginning with decade 9 (1990), our data show obvious changes. There are fewer taxa with an increase of records whereas there are considerably more taxa with a decrease of records. From decade 10 to 11 more taxa showed a decrease than an increase of records (Fig. 5).

In decade 10 (1990–1999) 22 species were no longer reported that were present in the previous decades. In decade 11 this figure increased to 42 species (see Appendix 2). Species that “disappeared” from Germany are e.g., *Ochrosisventralis* (Illiger, 1807) and *Psylliodesluteola* (Müller, 1776). However, records from other areas, e.g., the Czech Republic exist for both species (Čižek 2006). The most recent record of *Entomoscelisadonidis* (Pallas, 1771) in whole Central Europe, e.g., is of 1982. From decade 10 to decade 11 only 192 taxa were reported with increased record numbers. Of these taxa, only eight species with more than 250 records each contributed 3,509 records to the total number. In decade 11, we had records of 687 taxa in total. Of these, 486 (71%) were reported with fewer records than in decade 10.

## Discussion

Our database shows that the number of reported species decreased in the last decade although the total number of records increased (Fig. 4). This total increase of records is caused by only few highly abundant species. Our assessment suggests a decline in seed and leaf beetles in Central Europe since 1990. However, there are serious caveats: the continuous increase of records from 1900 to 2009 or 1900 to 2017, respectively (Figs 4, 2), reflects the activity of the collectors whose specimens are stored in the public collections we could exploit, and the motivation of those amateur collectors who reported their data to us or who published their findings. The activity of the amateur and professional collectors who contributed data varied over time and space. There are regions in Central Europe where entomological clubs are active whereas in others there are no such associations. Additionally, amateurs tend to collect in areas highly attractive to tourists, and where they expect a high diversity and abundance of the species in which they are interested. A major consequence is the inhomogeneous coverage of records over our study area (Fig. 1). Also, numerous collectors focus on certain subtaxa, sometimes even single genera, and ignore the remaining seed and leaf beetle species (see also Rheinheimer and Hassler 2018: 52). However, data on widespread and common species can also yield useful information on a possible biodiversity crisis (Conrad et al. 2006) but are probably underrepresented in our database. The collected specimens were identified to species or subspecies by taxonomists of different levels of expertise. Thus, our database likely contains some taxonomically incorrect records. During the past 20 years, a considerable number of leaf beetle taxonomists died, and only few younger taxonomists specialised on Chrysomelidae (E Geiser, Salzburg, pers. comm 2018, J Bezděk, Brno, pers. comm. 2019). As a consequence, the proportion of erroneous records probably increased because individuals of rare species were overlooked or incorrectly identified. This could in part explain the list of species with missing entries in ChryFaun since 1990 or 2000.

In the course of the last 100 years, the number of collected and reported seed and leaf beetles increased (Fig. 4). The number of reported species or subspecies increased more or less continuously from 1900 to 1999 but decreased markedly in the decade from 2000 to 2009. We conclude that there are fewer species of Chrysomelidae s.l. in Central Europe today than in 1990. Moreover, we consider the significant increase of the number of the species with decreased records during the last two decades of our study period as an indication of a serious threat to leaf beetle diversity. Quite a remarkable number of species has not been reported since 1990, and even more since 2000 (see Appendix 2). Even if we take into account that many of these “disappeared” species were or are rare and/or occur in areas from where we have only a limited number of records at all, we argue that the missing records are an alarming indication of a disappearance or even extinction in nature. Winkelman and Beenen (2010) found that a similar number of leaf beetle species had disappeared from the fauna of The Netherlands since 2000. Even some introduced stored-product pest species (marked with an * in Appendix 2) were no longer reported after 2000. We decided not to omit them from our list as this decrease of records might be indicative of factors that also influence the data on non-pest species.

Several authors, e.g., Thomas and Lennon (1999), Hickling et al. (2006), and Mason et al. (2015) discuss a general latitudinal expansion or shift of ranges of numerous invertebrate and vertebrate species as a consequence of global warming, as was found for the spider *Argiopebruennichi* (Scopoli, 1772) (Krehenwinkel and Tautz 2013). As to leaf beetles, only a surprisingly low number of species, 25 of the 246 analysed ones, meet our expectations. Our finding that 107 species now have a more eastern distribution as compared to the time period prior to 1980, and 18 more to north-east and 19 to south-east, must be seen with great reservations since the data coverage of the eastern part of Central Europe is low, so this effect is most likely due to a strong general increase in number of records for the east. Nevertheless, even here there may be a real natural process underlying our data.

Generally, oscillations of abundances within certain limits are natural and might vary from year to year. Temperature, precipitation, plant growth, food availability, but also diseases, parasites, and predators influence the number of individuals in a given area (Rheinheimer and Hassler 2018: 52). Above all, climate change, loss or fragmentation of habitats or their degradation are discussed in literature as possible causes of species declines and/or changes of range (Thomas et al. 2004; Köhler 2010; Kosior et al. 2007; Piper and Compton 2010; Hallmann et al. 2017). According to the European Environment Agency (Kurnik 2017), the average temperature in Europe increased between 2006 and 2015 by 1.45 to 1.59 °C as compared to the pre-industrial era. As the development and growth of ectothermic organisms like leaf beetles is strongly influenced by the ambient temperature, an increase in the number of records could reflect global warming. However, the observation that only 25 species extended their range towards north and 18 to north-east might suggest that global warming is probably not a major, or at least not a crucial, driver of range extensions of leaf beetles in Central Europe. The critical finding is that the number of species in our database decreased although the total number of records increased.

Loss and fragmentation of habitats are known to be responsible for the decline or even complete disappearance of species. Increasing mobility and economic activity, urbanisation, and expansion and change of agriculture are the drivers of changing landscapes (Opdam and Wascher 2004). Between 2000 and 2006 an area of 6256 km² was turned from green land into settlements and traffic zones in Germany (UBA1 2018). At the same time the German Environment Agency (Umweltbundesamt, UBA) reports a loss of 5278 km² of agricultural areas (fields and grassland, 1212 km² of forests and semi-natural areas, and 434 km² of wetlands (UBA1 2018). Additionally, the agriculture was intensified on the remaining areas (Gömann and Weingarten in press, Opdam and Wascher 2004). Hallmann et al. (2017) explained the decline of the biomass of flying insects in nature reserves by 75% over the past 27 years by these changes in agriculture. According to Kosior et al. (2007), the intensification of agriculture is responsible for the threat of 80% of the bumblebees and cuckoo bees in Western and Central Europe. The intensified forestry and agriculture is also a likely cause of the decline of butterflies (Warren et al. 2001) and moths (Conrad et al. 2006) in Great Britain.

Potts et al. (2010) state that the increased use of agrochemicals results in degradation of habitat quality. According to Biesmeijer et al. 2006, the application of agrochemicals caused a parallel decline of pollinating insects and insect-pollinated plants in The Netherlands and in Great Britain. We could not find data on the amount of pesticides applied in Europe. The German Environment Agency published only the national sales figures of the different types of pesticide products. These figures increased only minimally from 1995 to 2016 in pesticides for field crops (UBA2 2018).

In discussions on the possible causes of decline of species and biotope types, the authors of Red Lists agree that loss and fragmentation of habitats and changes in agriculture are the main driving factors (e.g., Korneck et al. 1998, Fritzlar 2011). Detailed analyses such as Heinig and Schoeller (2017) list manmade causes as the major factors of threat to leaf beetle diversity, e.g., increasing rarity of suitable habitats, lowering the groundwater table, and eutrophication of water bodies. Our observations point to the same direction: fewer than expected species extended their range towards the north, mono-, oligo- and polyphagous species are affected to similar degrees, and a remarkable increase of species with fewer records began with decade 8, i.e., from 1980. The above-mentioned suspected causes of insect decline have been known for a long time, as emphatically stated by Klausnitzer and Segerer (2018).

The usable habitats are fragmented like islands on which populations are trapped (Opdam and Wascher 2004). Species can react differently to fragmentation, with specialists suffering particularly (Kotze and O’Hara 2003; Biesmeijer et al. 2006; Nilsson et al. 2008). In contrast, our data do not show significant differences in de- and increase of record numbers from one quartile to the following in the species of the three trophic types. This could mean that specialists and generalists are affected in the same way and to similar degree by the factors causing insect decline.

Insects with low dispersal ability are less prone to escape from unfavourable habitat fragments in a landscape heavily modified by human activities (Warren et al. 2001; CD Thomas et al. 2004). The leaf beetle *Cryptocephalusnitidulus* Fabricius, 1787 is such a case (Piper and Compton 2010).

Loss and change of habitats are major factors influencing distribution and abundance of organisms (Hughes 2000) and have certainly also an impact in Chrysomelidae s.l. Our results are in concordance with numerous studies on insect decline over the past 30 years (e.g., Biesmeijer et al. 2006; Conrad et al. 2006; Hallmann et al. 2017; Kosior et al. 2007; Kotze and O’Hara 2003; Nilsson et al. 2008; Warren et al. 2001). However, such a parallelism is surprising because distribution and abundance of leaf beetles depend crucially on the availability of their food plants. Regrettably, data on changes of general plant distributions in Central Europe are not available.

The alarming news about the decline of insects of many different orders underpins the need for a continuous monitoring of their numbers and distribution. However, monitoring will only yield data from now on. For an analysis of past developments we do not have data meeting the standards of the monitoring (screening defined areas with identical sampling methods at regular intervals). Nevertheless, the fact that our results gained from the database ChryFaun (complete loss of ca. 6% of all species, decrease of records for 71% of all species since 2000) correspond to many other studies shows that data from private and museum collections can contribute to the analysis of insect decline. Such data are stored in numerous museum collections and even more in collections of amateurs, whose taxonomic expertise often excels that of museum curators (see Köhler 1997). It would be desirable to make collection data available for analyses of processes and possible causes of insect decline. Nevertheless, even taking all mentioned drawbacks into account, we are confident that the trends our results suggest are not mere artefacts but can be considered reliable proxies for real processes in nature.

## Appendix 1

**Table:** List of species and their change in distribution (N - north, E - east, S - south, W - west, NE - north-east, NW - north-west, SE - south-east, SW - south-west, Sh - shrinking, / - no change).

Types of geographical distributio (Schmitt and Rönn 2011)	Name of species	Change in distribution
Not specified	*Diabroticavirgifera* LeConte, 1858	/
	*Gonioctenaarctica* Mannerheim, 1853	Sh
	*Gonioctenanivosa* (Suffrian, 1851)	/
	*Oulemaseptentrionis* (Weise, 1880)	/
	*Phyllotretabalcanica* Heikertinger, 1909	/
	*Phyllotretaconsobrina* (Curtis, 1837)	/
Fewer than 10 records	*Gonioctenaflavicornis* (Suffrian, 1851)	/
	*Gonioctenakaufmanni* (Miller, 1880)	/
	*Gonioctenavariabilis* (Olivier, 1790)	/
	*Phyllotretaacutecarinata* Heikertinger, 1941	/
	*Phyllotretahochetlingeri* Fleischer, 1917	/
	*Phyllotretavariipennis* (Boieldieu, 1859)	/
	*Phyllotretaziegleri* Lohse, 1980	/
	*Timarchagibba* (Hagenbach, 1825)	/
	*Timarcharugulosa* Herrich-Schaeffer, 1838	/
	*Zeugophoraturneri* Power, 1863	/
Alpine	*Gonioctenaholdhausi* (Leeder, 1950)	/
Fragmented	*Phyllotretaatra* (Fabricius, 1775)	N, SE
	*Phyllotretacruciferae* (Goeze, 1777)	E, SE
	*Phyllotretastriolata* (Fabricius, 1803)	E, SE
Montane	*Aphthonaovata* Foudras, 1860	NW
	*Calomicrusgularis* (Gredler, 1857)	/
	*Chaetocnemaangustula* (Rosenhauer, 1847)	/
	*Chrysolinaaurichalcea* (Mannerheim, 1825)	/
	*Cryptocephalusnitidulus* Fabricius, 1787	E
	*Longitarsushelvolus* Kutschera, 1863	/
	*Luperusviridipennis* Germar, 1824	/
	*Luperusxanthopoda* (Schrank, 1781)	N, SE
	*Oreina alpestris* (Schummel, 1843)	E
	*Oreina bifrons* (Fabricius, 1792)	NE
	*Oreina cacaliae* (Schrank, 1785)	NE, SW
	*Oreina intricata* (Germar, 1824)	E
	*Oreina speciosa* (Linnaeus, 1767)	NE, Sh
	*Oreina speciosissima* (Scopoli, 1763)	NE
	*Psylliodesglabra* (Duftschmid, 1825)	N
	*Psylliodestoelgi* Heikertinger, 1914	/
	*Psylliodesvindobonensis* Heikertinger, 1914	/
	*Sclerophaedoncarniolicus* (Germar, 1824)	Sh
Northern	*Galerucellagrisescens* (Joannis, 1865)	E, S
	*Longitarsusplantagomaritimus* Dollman, 1912	E, W
	*Manturachrysanthemi* (Koch, 1803)	E, NW
	*Phaedonconcinnus* Stephens, 1831	S
	*Phyllotretaarmoraciae* (Koch, 1803)	N, E
	*Prasocurishannoverana* (Fabricius, 1775)	W
	*Psylliodescrambicola* Lohse, 1954	/
	*Psylliodesmarcida* (Illiger, 1807)	E, W
Eastern	*Colaphellussophiae* (Schaller, 1783)	E, Sh
	*Phyllotretascheuchi* Heikertinger, 1941	/
Southern	*Alticahelianthemi* (Allard, 1859)	S, Sh
	*Alticatamaricis* Schrank, 1785	/
	*Aphthonaabdominalis* (Duftschmid, 1825)	/
	*Aphthonaatrovirens* (Förster, 1849)	W
	*Aphthonacyparissiae* (Koch, 1803)	/
	*Aphthonaherbigrada* (Curtis, 1837)	W
	*Aphthonapallida* (Bach, 1856)	N
	*Aphthonapygmaea* (Kutschera, 1861)	E
	*Aphthonavenustula* (Kutschera, 1861)	/
	*Apteropedaorbiculata* (Marsham, 1802)	/
	*Derocrepisrufipes* (Linnaeus, 1758)	/
	*Hermaeophagamercurialis* (Fabricius, 1792)	NE, SE
	*Lachnaiasexpunctata* (Scopoli, 1763)	/
	*Neocrepidoderafemorata* (Gyllenhal, 1813)	N, E
	*Ochrosisventralis* (Illiger, 1807)	W, Sh
	*Phratoratibialis* (Suffrian, 1851)	N, E
	*Phratoravulgatissima* (Linnaeus, 1758)	E, S
	*Phyllotretanigripes* (Fabricius, 1775)	E, SE
	*Plagioderaversicolora* (Laicharting, 1781)	E
	*Sphaerodermarubidum* (Graëlls, 1858)	E, NE
	*Calomicruscircumfusus* (Marsham, 1802)	E
	*Calomicruspinicola* (Duftschmid, 1825)	N, W
	*Cassidapanzeri* Weise, 1907	E, Sh
	*Chaetocnemaarida* Foudras, 1860	E
	*Chaetocnemaobesa* (Boieldieu, 1859)	N, E
	*Chaetocnemasemicoerulea* (Koch, 1803)	E, Sh
	*Chrysolinacuprina* (Duftschmid, 1825)	/
	*Chrysolinahemisphaerica* (Germar, 1817)	N
	*Chrysolinaherbacea* (Duftschmid, 1825)	E, NE, SW
	*Chrysolinahyperici* (Forster, 1771)	E
	*Chrysolinamarginata* (Linnaeus, 1858)	E
	*Chrysolinarufa* (Duftschmid, 1825)	NE, Sh
	*Chrysomelacuprea* Fabricius, 1775	E, NE
	*Chrysomelasaliceti* (Weise, 1884)	E, Sh
	*Chrysomelavigintipunctata* (Scopoli, 1763)	E, NE
	*Coptocephalarubicunda* (Laicharting, 1781)	E
	*Crepidoderaaurea* (Geoffroy, 1785)	E
	*Crepidoderalamina* (Bedel, 1901)	/
	*Crepidoderanitidula* (Linnaeus, 1758)	E
	*Cryptocephalusbiguttatus* (Scopoli, 1763)	E
	*Cryptocephalusfrontalis* Marsham, 1802	/
	*Cryptocephaluslaetus* Fabricius, 1792	/
	*Cryptocephalusprimarius* Harold, 1872	/
	*Cryptocephaluspygmaeus* Fabricius, 1792	/
	*Cryptocephalusquerceti* Suffrian, 1848	E
	*Cryptocephalusquinquepunctatus* (Scopoli, 1763)	E, Sh
	*Cryptocephalussaliceti* Zebe, 1855	N, E
	*Cryptocephalusschaefferi* Schrank, 1789	SE
Southern	*Cryptocephalussexpunctatus* (Linnaeus, 1758)	E
	*Cryptocephalussignatifrons* Suffrian, 1847	N
	*Cryptocephalusvariegatus* Fabricius, 1781	/
	*Cryptocephalusvittatus* Fabricius, 1775	E
	*Diboliafoersteri* Bach, 1859	NW, SW, Sh
	*Donacia springeri* Müller, 1916	/
	*Epitrixatropae* Foudras, 1860	E
	*Epitrixintermedia* Foudras, 1860	/
	*Galerucalaticollis* (Sahlberg, 1837)	E
	*Galerucellatenella* (Linnaeus, 1760)	E
	*Gonioctenaintermedia* (Helliesen, 1913)	E, NW
	*Gonioctenalinnaeana* (Schrank, 1781)	SE, Sh
	*Gonioctenapallida* (Linnaeus, 1758)	NE
	*Gonioctenaviminalis* (Linnaeus, 1758)	E
	*Hispaatra* Linnaeus, 1767	E, NE
	*Labidostomishumeralis* (Schneider, 1792)	S
	*Labidostomislucida* (Germar, 1823)	N
	*Labidostomispallidipennis* (Gebler, 1839)	/
	*Labidostomistridentata* (Linnaeus, 1758)	E
	*Liliocerismerdigera* (Linnaeus, 1758)	/
	*Longitarsusabsynthii* Kutschera, 1862	/
	*Longitarsusechii* (Koch, 1803)	N, W
	*Longitarsuslateripunctatus* (Rosenhauer, 1856)	NW
	*Longitarsuslongiseta* Weise, 1889	/
	*Longitarsusmembranaceus* (Foudras, 1860)	/
	*Longitarsusminusculus* (Foudras, 1860)	/
	*Longitarsusnanus* (Foudras, 1860)	SW
	*Longitarsuspellucidus* (Foudras, 1860)	E
	*Longitarsuspulmonariae* Weise, 1893	N, E
	*Longitarsusscutellaris* (Rey, 1873)	Sh
	*Luperusflaviceps* Apfelbeck, 1912	E
	*Manturamathewsi* (Curtis, 1833)	/
	*Oomorphusconcolor* (Sturm, 1807)	N, E
	*Orsodacnecerasi* (Linnaeus, 1758)	N, E
	*Pachnephoruspilosus* (Rossi, 1790)	/
	*Pachybrachishieroglyphicus* (Laicharting, 1781)	E
	*Pachybrachishippophaes* Suffrian, 1848	/
	*Pachybrachispicus* Weise, 1882	Sh
	*Pachybrachissinuatus* Mulsant, 1859	E
	*Pachybrachistessellatus* (Olivier, 1791)	E, SE
	*Phaedonlaevigatus* (Duftschmid, 1825)	NW
	*Phyllotretachristinae* Heikertinger, 1941	E, NW
	*Phyllotretaochripes* (Curtis, 1837)	N, E
	*Phyllotretaprocera* (Redtenbacher, 1849)	S, SE
	*Phyllotretapunctulata* (Marsham, 1802)	/
	*Prasocurisglabra* (Herbst, 1783)	E
	*Psylliodeschalcomera* (Illiger, 1807)	E, W
	*Psylliodesinstabilis* Foudras, 1860	/
	*Psylliodesisatidis* Heikertinger, 1913	E, S
	*Psylliodesthlaspis* Foudras, 1860	/
Southern	*Smaragdinaaffinis* (Illiger, 1794)	SE, Sh
	*Smaragdinaflavicollis* (Charpentier, 1825)	NE
	*Timarchagoettingensis* (Linnaeus, 1758)	E, Sh
	*Timarchametallica* (Laicharting, 1781)	Sh
	*Timarchapratensis* (Duftschmid, 1825)	/
	*Zeugophorafrontalis* Suffrian, 1840	/
South-Eastern	*Bruchidiusmarginalis* (Fabricius, 1776)	/
	*Bruchusatomarius* (Linnaeus, 1761)	N, E
	*Cassidaferruginea* Goeze, 1777	/
	*Cassidarufovirens* Suffrian, 1844	N
	*Cassidasanguinolenta* Müller, 1776	E
	*Cassidasubferruginea* (Schrank, 1776)	/
	*Cassidasubreticulata* Suffrian, 1844	N, E
	*Cassidavibex* Linnaeus, 1767	E
	*Chrysochusasclepiadeus* (Pallas, 1773)	/
	*Chrysolinageminata* (Paykull, 1799)	/
	*Chrysolinakuesteri* (Helliesen, 1912)	E
	*Chrysolinalichenis* (Richter, 1820)	NE
	*Chrysolinasturmi* (Westhoff, 1882)	E
	*Chrysolinavarians* (Schaller, 1783)	E
	*Chrysomelapopuli* Linnaeus, 1758	E
	*Chrysomelatremula* Fabricius, 1783	SE
	*Clytralaeviuscula* Ratzeburg, 1837	E, Sh
	*Clytraquadripunctata* (Linnaeus, 1758)	E
	*Coptocephalaunifasciata* (Scopoli, 1763)	E
	*Cryptocephalusaureolus* Suffrian, 1847	E, NE
	*Cryptocephalusbilineatus* (Linnaeus, 1767)	E
	*Cryptocephaluschrysopus* Gmelin, 1788	E, W, Sh
	*Cryptocephaluscordiger* (Linnaeus, 1758)	SE
	*Cryptocephaluselegantulus* Gravenhorst, 1807	SE
	*Cryptocephalusexiguus* Schneider, 1792	S
	*Cryptocephalusfrenatus* Laicharting, 1781	/
	*Cryptocephalushypochaeridis* (Linnaeus, 1758)	E
	*Cryptocephalusmarginatus* Fabricius, 1781	/
	*Cryptocephalusmoraei* (Linnaeus, 1758)	E
	*Cryptocephalusoctopunctatus* (Scopoli, 1763)	N, E
	*Cryptocephalusviolaceus* Laicharting, 1781	SE
	*Cryptocephalusvittula* Suffrian, 1848	/
	*Diboliadepressiuscula* (Letzner, 1847)	/
	*Diboliafemoralis* Redtenbacher, 1849	W
	*Diboliarugulosa* Redtenbacher, 1849	SE. Sh
	*Galerucatanaceti* (Linnaeus, 1758)	E
	*Gonioctenafornicata* (Brüggemann, 1873)	W
	*Gonioctenagobanzi* (Reitter, 1902)	E
	*Labidostomiscyanicornis* (Germar, 1817)	Sh
	*Labidostomislongimana* (Linnaeus, 1761)	E
	*Longitarsusapicalis* (Beck, 1817)	/
	*Longitarsusballotae* (Marsham, 1802)	E
	*Longitarsusfoudrasi* Weise, 1893	N
	*Longitarsusmelanocephalus* (De Geer, 1775)	E
South-Eastern	*Longitarsusnigrofasciatus* (Goeze, 1777)	N, E
	*Longitarsusobliteratus* (Rosenhauer, 1847)	NE, NW
	*Longitarsussalviae* Gruev, 1975	/
	*Manturaobtusata* (Gyllenhal, 1813)	/
	*Minotaobesa* (Waltl, 1839)	/
	*Oulemaobscura* (Stephens, 1831)	E, W
	*Phyllotretadiademata* Foudras, 1860	E, Sh
	*Phyllotretanodicornis* (Marsham, 1802)	/
	*Podagricafuscicornis* (Linnaeus, 1767)	E, Sh
	*Smaragdinaaurita* (Linnaeus, 1767)	E
	*Smaragdinasalicina* (Scopoli, 1763)	E
South-East	*Phyllotretaganglbaueri* Heikertinger, 1909	/
South-Western	*Apteropedaglobosa* (Illiger, 1794)	N
	*Apteropedasplendida* Allard, 1860	/
	*Bruchusoccidentalis* Lukjanovitsh & Ter-Minassian, 1957	/
	*Cryptocephalusocellatus* Drapiez, 1819	E
	*Diboliacryptocephala* (Koch, 1803)	E
	*Donacia bicolora* Zschach, 1788	/
	*Donacia simplex* Fabricius, 1775	/
	*Longitarsusaeruginosus* (Foudras, 1860)	E, S
	*Longitarsusganglbaueri* Heikertinger, 1912	/
	*Longitarsusrubiginosus* (Foudras, 1860)	E, W
	*Mniophilamuscorum* (Koch, 1803)	SW
	*Timarchatenebricosa* (Fabricius, 1775)	/
Unusual	*Gonioctenadecemnotata* (Marsham, 1802)	E, W
	*Gonioctenainterposita* (Franz & Palmén, 1950)	E, NW, Sh
	*Gonioctenaolivacea* (Forster, 1771)	E, S
	*Orsodacnehumeralis* Latreille, 1804	E, W
	*Oulemaduftschmidi* (Redtenbacher, 1874)	E, S
	*Phyllotretaastrachanica* Lopatin, 1977	NE
	*Phyllotretaaustriaca* Heikertinger, 1909	/
	*Prasocurisjunci* (Brahm, 1790)	E
Scattered	*Cryptocephalusquadripustulatus* Gyllenhal, 1813	E
	*Oulemaerichsonii* (Suffrian, 1841)	/
	*Phyllotretadilatata* Thomson, 1866	E, NW
	*Phyllotretaflexuosa* (Illiger, 1794)	E, W
Wide	*Chrysolinacoerulans* (Scriba, 1791)	E
	*Cryptocephaluscoryli* (Linnaeus, 1758)	/
	*Gonioctenaquinquepunctata* (Fabricius, 1787)	E, NE
	*Leptinotarsadecemlineata* (Say, 1824)	E
	*Liliocerislilii* (Scopoli, 1763)	/
	*Oulemamelanopus* (Linnaeus, 1758)	E, S
	*Phyllotretanemorum* (Linnaeus, 1758)	E, SE
	*Phyllotretaexclamationis* (Thunberg, 1784)	E
	*Phyllotretatetrastigma* (Comolli, 1837)	/
	*Phyllotretaundulata* Kutschera, 1860	SE
	*Phyllotretavittula* (Redtenbacher, 1849)	E, SE
	*Zeugophoraflavicollis* (Marsham, 1802)	/
	*Zeugophorascutellaris* Suffrian, 1840	/
	*Zeugophorasubspinosa* (Fabricius, 1781)	E

## Appendix 2

**Table:** List of species and subspecies that were not reported after 1990 (decade 10) or after 2000 (decade 11). Species marked with an asterisk (*) are pests of stored products, species marked ** were reported informally, but we have no label data in ChryFaun.

Since	Name of species
1990	*Aphthonaaeneomicans* Allard, 1875
	*Argopusbicolor* Fischer, 1824
	*Cassidainquinata* Brullé, 1832
	*Chrysolinadidymata* (Scriba, 1791)
	*Cryptocephalusbimaculatus* Fabricius, 1781
	*Cryptocephalusbohemius* Drapiez, 1819
	*Cryptocephaluscyanipes* Suffrian, 1847
	*Cryptocephalusgridellii* Burlini, 1950**
	*Cryptocephalusloreyi* Solier, 1836
	*Entomoscelisadonidis* (Pallas, 1771)**
	*Epitrixintermedia* Foudras, 1860
	*Gonioctenagobanzi* (Reitter, 1902)
	*Longitarsuscizeki* Döberl, 2004
	*Neocrepidoderabasalis* (K Daniel, 1900)
	*Oreina peirolerii* (Bassi, 1834)
	*Orestiaheikertingeri* Leonardi, 1974
	Prasocuris (Hydrotassa) flavocincta (Brullé, 1832)
	*Psylliodesgibbosa* Allard, 1860
	*Psylliodeskiesenwetteri* Kutschera, 1864
	*Psylliodesluteola* (Müller, 1776)
	*Stylosomusilicicola* Suffrian, 1848
	*Timarchanicaeensis* (Villa & Villa, 1835)
2000	*Aphthonailligeri* Bedel, 1898
	*Aphthonastussineri* Weise, 1888
	*Bruchidiusdispar* (Gyllenhal, 1833)
	*Bruchidiuslividimanus* (Gyllenhal, 1833)
	*Bruchidiusmartinezi* (Allard, 1868) – probably incorrect identification
	*Bruchusgriseomaculatus* Gyllenhal, 1833
	*Bruchussibiricus* Germar, 1824 – probably incorrect identification
	*Bruchusvenustus* Fahraeus, 1839
	*Callosobruchuschinensis* (Linnaeus, 1758)*
	*Caryedonserratus* (Olivier, 1790)*
	*Chaetocnemachlorophana* (Duftschmid, 1825)
	*Chaetocnemamajor* (Jacquelin-Duval, 1852)
	*Chrysolinaamericana* (Linnaeus, 1758)
	*Chrysolinaasclepiadisasclepiadis* (Villa & Villa, 1833)
	*Chrysolinafimbrialis* (Kuester, 1845)
	*Chrysolinaglobosa* (Panzer, 1805)
	*Chrysolinagrossa* (Fabricius, 1792)
	*Chrysolinaolivieri* (Bedel, 1892)
	*Chrysolinarelucens* (Rosenhauer, 1847)
	*Chrysolinarufoaenea* (Suffrian, 1851)
	*Chrysolinaschneideri* (Weise, 1882)**
	*Chrysolinacarpathica* (Fuss, 1856)**
	*Coptocephalachalybaea* (Germar, 1824)**
	*Cryptocephaluslaevicollis* Gebler, 1830**
2000	*Cryptocephalusplanifrons* Weise, 1882**
	*Cryptocephalusquatuordecimmaculatus* Schneider, 1792**
	*Cryptocephalustransiens* Franz, 1949**
	*Cryptocephalusvirens* Suffrian, 1847**
	*Derocrepissodalis* (Kutschera, 1860)
	*Entomoscelissacra* (Linnaeus, 1758)**
	*Galerucajucunda* (Faldermann, 1837)
	*Gonioctenakaufmanni* (Miller, 1880)**
	*Gonioctenavariabilis* (Olivier, 1790) – probably incorrect identification
	*Labidostomispallidipennis* (Gebler, 1839)
	*Lachnaiaitalica* (Weise, 1882)
	*Longitarsusweisei* Guillebeau, 1895
	*Luperusnigripes* Kiessenwetter, 1861**
	*Minotaalpina* Biondi, 1986
	*Minotaimpuncticollis* (Allard, 1860)
	*Neocrepidoderaadelinae* (Binaghi, 1947)
	*Neocrepidoderacyanipennis* (Kutschera, 1860)
	*Neocrepidoderaobirensis* (Ganglbauer, 1897)
	*Neocrepidoderasimplicipes* (Kutschera, 1860)
	*Oreina liturata* (Scopoli, 1763)
	*Orestiaelectra* Gredler, 1868
	*Phyllobroticaadusta* (Creutzer, 1799)
	*Phyllotretaconsobrina* (Curtis, 1837)
	*Phyllotretaziegleri* Lohse, 1980
	*Psylliodesdanieli* Weise, 1900
	*Psylliodesrambousekiforojulensis* Heikertinger, 1926
	*Psylliodessubaeneastyriaca* Heikertinger, 1921
	*Smaragdinadiversipes* Letzner, 1839**
	*Zabrotessubfasciatus* (Boheman, 1833)*
